# Supplementary material for: Oyster Shell Powder, Zeolite and Red Mud as Binders for Immobilising Toxic Metals in Fine Granular Contaminated Soils (from Industrial Zones in South Korea)
Source: Int J Environ Res Public Health. 2021 Mar 4;18(5):2530. doi: 10.3390/ijerph18052530 (PMC7967652; doi:10.3390/ijerph18052530)
Supplement: Supplementary file 1 [file ijerph-18-02530-s001.pdf]

Supplementary Materials

Table S1. Chemical compositions of the silty sand soil, sandy soil and HCS; the binders, namely, oyster shell (OS), zeolite (Z) and red mud (RM) observed via X-ray fluorescence analysis.

	Silty sand soil	Sandy soil	HCS	OS	Z	RM
SiO_2	50.46	67.35	53.62	0.240	70.75	15.18
Al_2O_3	19.01	18.79	24.95	0.013	13.93	17.47
Fe_2O_3	7.98	3.34	8.41	0.033	2.75	39.47
CaO	10.68	2.96	2.33	94.59	1.61	1.99
MgO	2.81	1.36	1.51	0.550	0.91	0.23
MnO	0.34	0.12	0.12	0.007	0.102	0.04
ZnO	0.48	0.07	0.02	-	-	-
Na_2O	1.66	1.46	1.81	1.100	3.95	10.46
K_2O	3.17	3.62	3.66	0.040	3.17	0.06
P_2O_5	0.89	0.20	0.70	0.110	0.105	0.12
TiO_2	1.54	0.61	1.06	0	0.38	6.85
Cr_2O_5	0.34	-	-	-	-	-
SO_3	0.64	0.11	-	-	-	-
PbO	-	-	1.11	-	-	-
Cl	-	-	0.44	-	-	-
Others			0.26	3.317	2.343	8.14

Table S2. pH value of the fine granular contaminated soils considered in this study after the addition of binders (OS, Z and RM) at various dosages.

Source	pH	Soil + Binder	1%	3%	5%	7.5%	10%
Abandoned metal mine site (silty sand soil)	8.13 ± 0.16	+ OS	7.95 ± 0.08	7.87 ± 0.06	8.01 ± 0.17		
		+ Z	7.75 ± 0.15	7.67 ± 0.16	7.62 ± 0.19		
		+ RM	7.96 ± 0.07	8.40 ± 0.21	9.03 ± 0.09	-	-
Military service area (sandy soil)	6.70 ± 0.15	+ OS	7.59 ± 0.20	7.78 ± 0.16	7.68 ± 0.36		
		+ Z	6.37 ± 0.12	6.44 ± 0.14	6.46 ± 0.12		
		+ RM	8.04 ± 0.14	8.90 ± 0.12	9.44 ± 0.04	-	-
HCS	4.90 ± 0.15	+ OS	7.31 ± 0.40	7.88 ± 0.12	7.65 ± 0.08	7.76 ± 0.27	7.50 ± 0.01
		+ Z	4.47 ± 0.05	4.59 ± 0.11	4.73 ± 0.04	4.94 ± 0.01	5.17 ± 0.01
		+ RM	6.24 ± 0.01	8.13 ± 0.18	8.62 ± 0.01	9.13 ± 0.10	9.61 ± 0.02

Table S3. pH value of the fine granular contaminated soils considered in this study treated with binders after leaching test via TCLP was performed.

Source	Soil + Binder	1%	3%	5%	7.5%	10%
Abandoned metal mine site (silty sand soil)	+ OS	5.22 ± 0.25	5.59 ± 0.38	6.78 ± 0.15		
	+ Z	5.12 ± 0.21	5.00 ± 0.10	5.01 ± 0.08		
	+ RM	4.99 ± 0.08	5.05 ± 0.04	4.99 ± 0.08		
Military service area (sandy soil)	+ OS	3.81 ± 0.06	4.48 ± 0.32	5.61 ± 1.16		
	+ Z	3.28 ± 0.02	5.00 ± 0.10	5.01 ± 0.08		
	+ RM	3.60 ± 0.06	3.92 ± 0.04	4.10 ± 0.02		
HCS	+ OS	5.18 ± 0.01	5.42 ± 0.03	6.35 ± 0.06	7.36 ± 0.06	7.54 ± 0.01
	+ Z	4.47 ± 0.01	4.52 ± 0.08	4.77 ± 0.11	4.88 ± 0.11	5.28 ± 0.05
	+ RM	4.75 ± 0.18	5.38 ± 0.11	6.80 ± 0.12	7.25 ± 0.08	7.98 ± 0.05

Table S4. Initial toxic metal concentrations in the considered soil samples and relevant permissible and guideline limits.

Country & Criterium	US<br>Leaching from waste	US<br>Surface water, fresh water	China<br>Soil Solution	China<br>*simulation of water runoff (with DI)	South Korea<br>Leaching from waste	Japan<br>Soil Solution	Canada<br>Soil Quality	WHO<br>Unpolluted Soil	South Korea<br>In Soil	Australia<br>In Soil	Canada<br>In Soil	European Union<br>In Soil
	(mg/L)						(mg/kg)					
Cd	1	2	1	2	0.3	0.01	22	0.8	10	3	1.4	10
Pb	5	2.5	5	0.1	3	0.01	600	85	400	300	70	200
Zn		120	100	0.01			410	50	600	200	200	250
Cu		9			3		91	36	500	100	63	150
Ni		52					89	35	200	60	50	100
Source	(U.S. EPA 2011)	(Buchman 2008)	(MEPC 2007)	(MEPC 2007)	(EHTI 2017)	(Liu et al. 2018)	(CCME 2020)	(WHO 1996)	(EHTI 2017)	(Liu et al. 2018)	(Liu et al. 2018)	(Liu et al. 2018)

* Soil Environmental Quality Standards in China (GB 5085.3-2007)

Table S5. Descriptive statistics of toxic metal concentrations obtained via TCLP test from the leachate of silty sand soil treated with OS, Z and RM.

		Cu				Zn				Cd				Ni				Pb			
		IC	+OS1	+OS3	+OS5	IC	+OS1	+OS3	+OS5	IC	+OS1	+OS3	+OS5	IC	+OS1	+OS3	+OS5	IC	+OS1	+OS3	+OS5
**S + OS**	*n* Samples	19	11	15	10	5	5	2	3	12	5	12	7	6	6	12	7	15	10	16	10
	Max	15.556	10.369	7.488	2.179	145.000	145.014	84.128	83.213	0.383	0.382	0.321	0.235	1.222	0.849	0.287	0.277	1.929	1.549	0.376	0.165
	Min	0.921	0.662	0.458	0.434	81.851	68.797	63.324	59.828	0.255	0.309	0.143	0.143	0.152	0.160	0.089	0.066	0.107	0.092	0.036	0.046
	Mean	3.954	3.191	1.506	0.937	102.784	94.013	73.726	68.657	0.316	0.349	0.224	0.182	0.432	0.313	0.158	0.136	0.639	0.403	0.149	0.084
	Std Dev	4.233	2.934	1.813	0.536	24.509	30.231	14.711	12.700	0.043	0.029	0.044	0.033	0.403	0.268	0.070	0.079	0.531	0.420	0.090	0.034
	Var	17.920	8.609	3.287	0.287	600.689	913.908	216.410	161.301	0.002	0.001	0.002	0.001	0.162	0.072	0.005	0.006	0.282	0.177	0.008	0.001
			+Z1	+Z3	+Z5		+Z1	+Z3	+Z5		+Z1	+Z3	+Z5		+Z1	+Z3	+Z5		+Z1	+Z3	+Z5
S + Z	*n* Samples		8	9	10		3	3	2		4	7	7		8	12	12		8	9	8
	Max		2.896	2.797	3.709		99.548	91.765	82.867		0.332	0.334	0.356		1.153	0.424	0.301		0.415	0.502	0.337
	Min		0.894	0.899	0.948		98.852	65.273	75.031		0.298	0.276	0.237		0.162	0.128	0.134		0.173	0.171	0.145
	Mean		1.736	1.679	1.906		99.153	76.822	78.949		0.322	0.306	0.289		0.418	0.215	0.204		0.273	0.285	0.230

	Std Dev	1.607	1.472	1.769	0.357	13.568	5.541	0.016	0.020	0.044	0.367	0.086	0.061	0.086	0.106	0.056
	Var	0.733	0.629	0.817	0.128	184.098	30.699	0.000	0.000	0.002	0.135	0.007	0.004	0.007	0.011	0.003
S + RM		+RM1	+RM3	+RM5	+RM1	+RM3	+RM5	+RM1	+RM3	+RM5	+RM1	+RM3	+RM5	+RM1	+RM3	+RM5
	n Samples	6	4	6	2	2	3	6	4	4	6	4	4	6	4	2
	Max	7.489	2.809	3.248	122.506	86.245	80.009	0.490	0.423	0.414	0.472	0.291	0.149	0.683	0.616	0.372
	Min	1.965	1.299	1.151	100.054	60.482	66.197	0.287	0.261	0.275	0.109	0.059	0.014	0.258	0.169	0.239
	Mean	4.140	2.004	2.030	111.280	73.363	71.551	0.386	0.340	0.348	0.260	0.168	0.115	0.449	0.373	0.306
	Std Dev	2.139	0.619	0.739	15.876	18.217	7.410	0.077	0.072	0.064	0.128	0.098	0.067	0.167	0.215	0.094
	Var	4.575	0.384	0.546	252.062	331.854	54.913	0.006	0.005	0.004	0.016	0.010	0.004	0.028	0.046	0.009

S – Soil sample; IC – Initial concentration; OS – Oyster shell; Z – zeolite; RM – Red mud. The number after the binder abbreviation represents mass percentage (e.g. Z3 means 3 wt% of Z).

Table S6. Variation in toxic metal concentrations in the leachate obtained via TCLP from sandy soil treated with OS, Z and RM.

		**Cu**				**Zn**				**Cd**				**Pb**			
**S + OS**		IC	+OS1	+OS3	+OS5	IC	+OS1	+OS3	+OS5	IC	+OS1	+OS3	+OS5	IC	+OS1	+OS3	+OS5
	n Samples	3	3	3	3	3	3	3	3	3	3	3	2	3	3	3	3
	Max	2.408	1.459	0.859	0.471	14.000	9.913	4.317	7.442	0.050	0.033	0.029	0.025	0.095	0.047	0.028	0.019
	Min	2.083	0.999	0.545	0.128	7.602	6.092	3.594	2.459	0.039	0.031	0.024	0.020	0.065	0.032	0.018	0.005
	Mean	2.235	1.203	0.725	0.298	10.053	7.648	3.858	4.956	0.046	0.032	0.026	0.023	0.079	0.039	0.022	0.012
	Std Dev	0.163	0.234	0.162	0.172	3.451	2.007	0.399	2.492	0.006	0.001	0.002	0.004	0.015	0.007	0.006	0.007
	Var	0.027	0.055	0.026	0.030	11.913	4.027	0.159	6.209	0.000	1.8E-06	4.4E-06	1.3E-05	2.3E-04	5.2E-05	3.3E-05	4.4E-05
**S + Z**			+Z1	+Z3	+Z5		+Z1	+Z3	+Z5		+Z1	+Z3	+Z5		+Z1	+Z3	+Z5
	n Samples		4	4	2		4	4	4		4	4	4		4	4	3
	Max		3.892	1.607	1.715		13.559	11.271	7.776		0.032	0.035	0.035		0.063	0.130	0.082
	Min		1.243	1.080	1.529		3.588	4.530	5.713		0.029	0.032	0.031		0.053	0.045	0.050
	Mean		2.000	1.251	1.622		6.799	6.419	6.302		0.030	0.034	0.033		0.058	0.068	0.061
	Std Dev		1.267	0.244	0.132		4.664	3.256	0.986		0.001	0.001	0.002		0.004	0.042	0.018
	Var		1.606	0.060	0.017		21.755	10.604	0.972		1.6E-06	1.8E-06	3.3E-06		1.8E-05	1.7E-03	3.1E-04
**S + RM**			+RM1	+RM3	+RM5		+RM1	+RM3	+RM5		+RM1	+RM3	+RM5		+RM1	+RM3	+RM5
	n Samples		2	3	3		3	3	3		3	3	3		3	3	3
	Max		1.957	1.513	1.677		14.592	15.847	8.907		0.044	0.030	0.028		0.127	0.108	0.132
	Min		1.751	1.306	1.229		8.139	5.227	5.213		0.038	0.028	0.023		0.077	0.071	0.117
	Mean		1.854	1.420	1.397		10.590	10.096	7.617		0.041	0.029	0.025		0.096	0.090	0.124

Std Dev	0.145	0.105	0.244	3.494	5.364	2.084	0.003	0.001	0.002	0.027	0.018	0.008
Var	0.021	0.011	0.060	12.211	28.777	4.343	1.1E-05	1.4E-06	4.6E-06	7.1E-04	3.2E-04	6.2E-05

S – Soil sample; IC – Initial concentration; OS – Oyster shell; Z – zeolite; RM – Red mud. The number after the binder abbreviation represents mass percentage (e.g. Z3 means 3 wt% of Z).

Table S7. Descriptive statistics of the toxic metal concentration for the leachate of HCS obtained from the TCLP test.

		**Cu**						**Pb**					
		HCS	+OS1	+OS3	+OS5	+OS7.5	+OS10	HCS	+OS1	+OS3	+OS5	+OS7.5	+OS10
HCS + OS	N Samples	9	4	2	2	2	2	4	2	2	2	2	2
	Max	0.48	0.48	0.40	0.35	0.28	0.19	175.56	299.71	321.86	295.15	215.02	144.63
	Min	0.39	0.27	0.33	0.28	0.24	0.16	145.46	286.38	263.49	244.73	202.82	139.91
	Mean	0.444	0.360	0.368	0.315	0.262	0.174	159.802	293.048	292.673	269.942	208.917	142.268
	Std Dev	0.03	0.09	0.05	0.05	0.03	0.02	12.87	9.42	41.27	35.66	8.63	3.34
	Var	0.00	0.01	0.00	0.00	0.00	0.00	165.71	88.83	1703.51	1271.35	74.44	11.15
			+Z1	+Z3	+Z5	+Z7.5	+Z10		+Z1	+Z3	+Z5	+Z7.5	Z10
HCS + Z	N Samples		2	2	2	2	2		2	2	2	2	2
	Max		0.26	0.19	0.22	0.20	0.17		164.61	120.78	144.40	133.76	109.28
	Min		0.21	0.16	0.18	0.19	0.15		152.47	114.16	83.25	132.56	108.53
	Mean		0.234	0.174	0.199	0.194	0.161		158.540	117.467	113.825	133.161	108.907
	Std Dev		0.03	0.02	0.03	0.00	0.02		8.59	4.68	43.24	0.85	0.53
	Var		0.00	0.00	0.00	0.00	0.00		73.71	21.90	1870.00	0.72	0.28
			+RM1	+RM3	+RM5	+RM7.5	+RM10	***IC ****	+RM1	+RM3	+RM5	+RM7.5	+RM10
HCS + RM	N Samples		2	2	2	2	2	***6***	2	2	2	2	2
	Max		0.27	0.26	0.22	0.17	0.20	***366.99***	233.18	225.13	193.65	149.88	125.03
	Min		0.25	0.23	0.19	0.17	0.15	***222.93***	220.05	197.36	161.63	146.14	124.25
	Mean		0.260	0.243	0.209	0.171	0.172	***301.657***	226.612	211.245	177.637	148.011	124.644
	Std Dev		0.02	0.02	0.02	0.00	0.04	***58.40***	9.29	19.63	22.64	2.64	0.55
	Var		0.00	0.00	0.00	0.00	0.00	***3410.17***	86.24	385.50	512.62	6.99	0.30

(* **bold**) The values shown in blue represent the results of the leaching test with DI water (initial concentrations). They are presented to provide an improved understanding of binder performance. OS – Oyster shell; Z – zeolite; RM – Red mud. The number after the binder abbreviation represents mass percentage.

Table S8. Descriptive statistics of the toxic metal concentration for the leachate of HCS obtained from the TCLP test when the supernatant was extracted at different times after treatment with oyster shell.

		Cu						Pb					
		HCS	+OS1	+OS3	+OS5	+OS7.5	+OS10	HCS	+OS1	+OS3	+OS5	+OS7.5	+OS10
**HCS + OS (1day)**	*n* Samples	9	4	4	4	2	2	4	2	2	2	2	2
	Max	0.48	0.63	0.35	0.10	0.02	0.01	175.56	324.58	150.25	39.85	8.51	4.05
	Min	0.39	0.33	0.25	0.04	0.02	0.01	145.46	183.93	118.13	37.68	7.57	3.27
	Mean	0.444	0.444	0.306	0.070	0.019	0.008	159.802	254.253	134.189	38.769	8.039	3.662
	Std Dev	0.03	0.13	0.05	0.03	0.00	0.00	12.87	99.45	22.71	1.53	0.66	0.55
	Var	0.00	0.02	0.00	0.00	0.00	0.00	165.71	9890.64	515.75	2.35	0.44	0.31
			+OS1	+OS3	+OS5	+OS7.5	+OS10		+OS1	+OS3	+OS5	+OS7.5	+OS10
**HCS + OS (10 days)**	*n* Samples		4	4	4	2	2		2	2	2	2	2
	Max		0.38	0.41	0.06	0.01	0.01		167.78	122.80	18.98	5.44	3.57
	Min		0.23	0.15	0.00	0.01	0.01		129.49	91.82	16.23	5.13	3.48
	Mean		0.322	0.280	0.033	0.011	0.007		148.637	107.310	17.605	5.283	3.524
	Std Dev		0.07	0.12	0.03	0.00	0.00		27.08	21.90	1.95	0.22	0.06
	Var		0.00	0.02	0.00	0.00	0.00		733.29	479.63	3.80	0.05	0.00

OS – Oyster shell. The number after the binder abbreviation represents mass percentage.

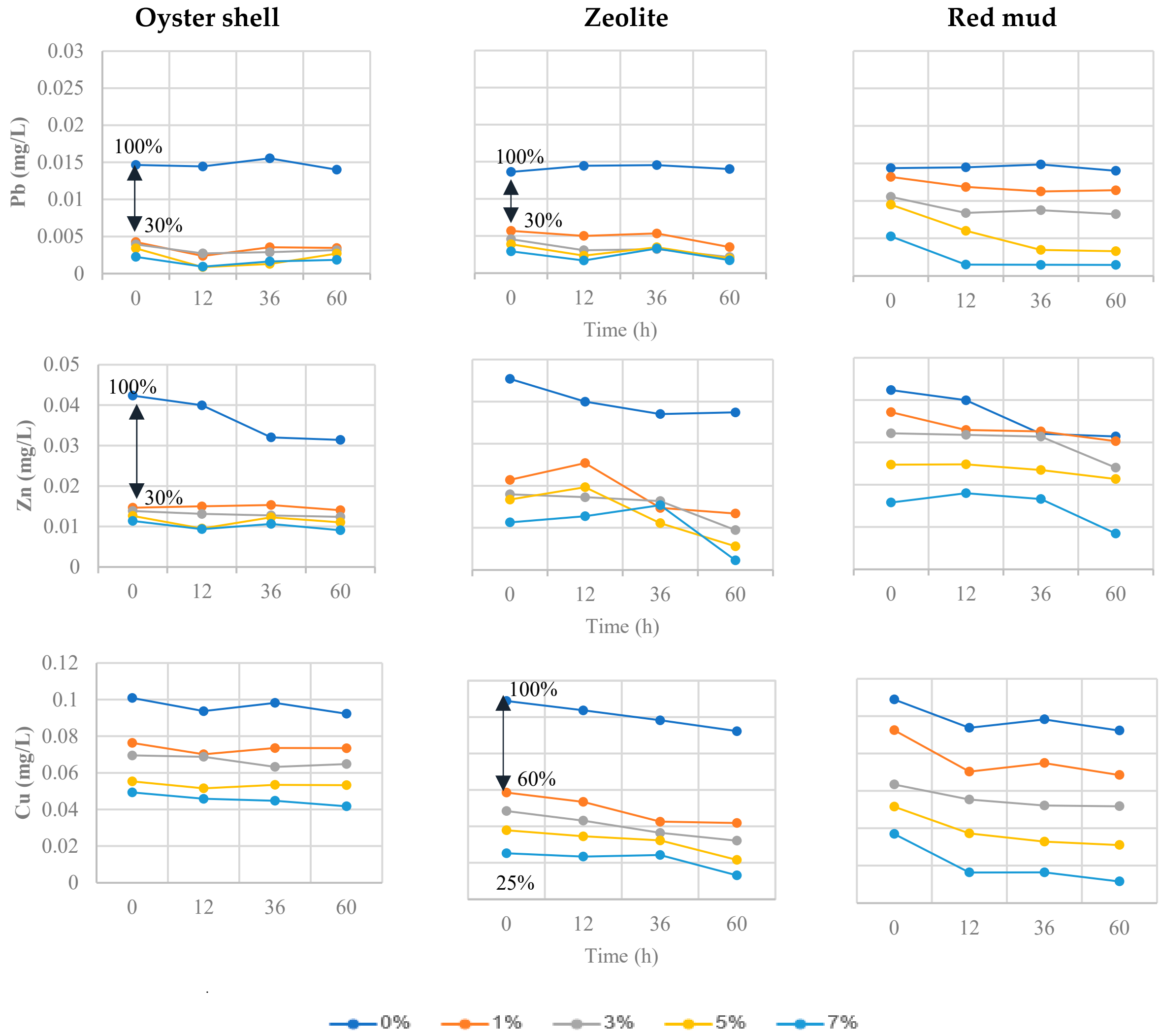

Figure S1. Mean toxic metal concentrations measured in the leachate after mixing the binder with the silty sand soil from an abandoned metal mine site (Source: 1st year report of project 'grant no. 2019002470002'). S – Soil sample; IC – Initial concentration; OS – Oyster shell; Z – zeolite; RM – Red mud. The number after the binder abbreviation represents mass percentage (e.g. Z3 means 3 wt% of Z).

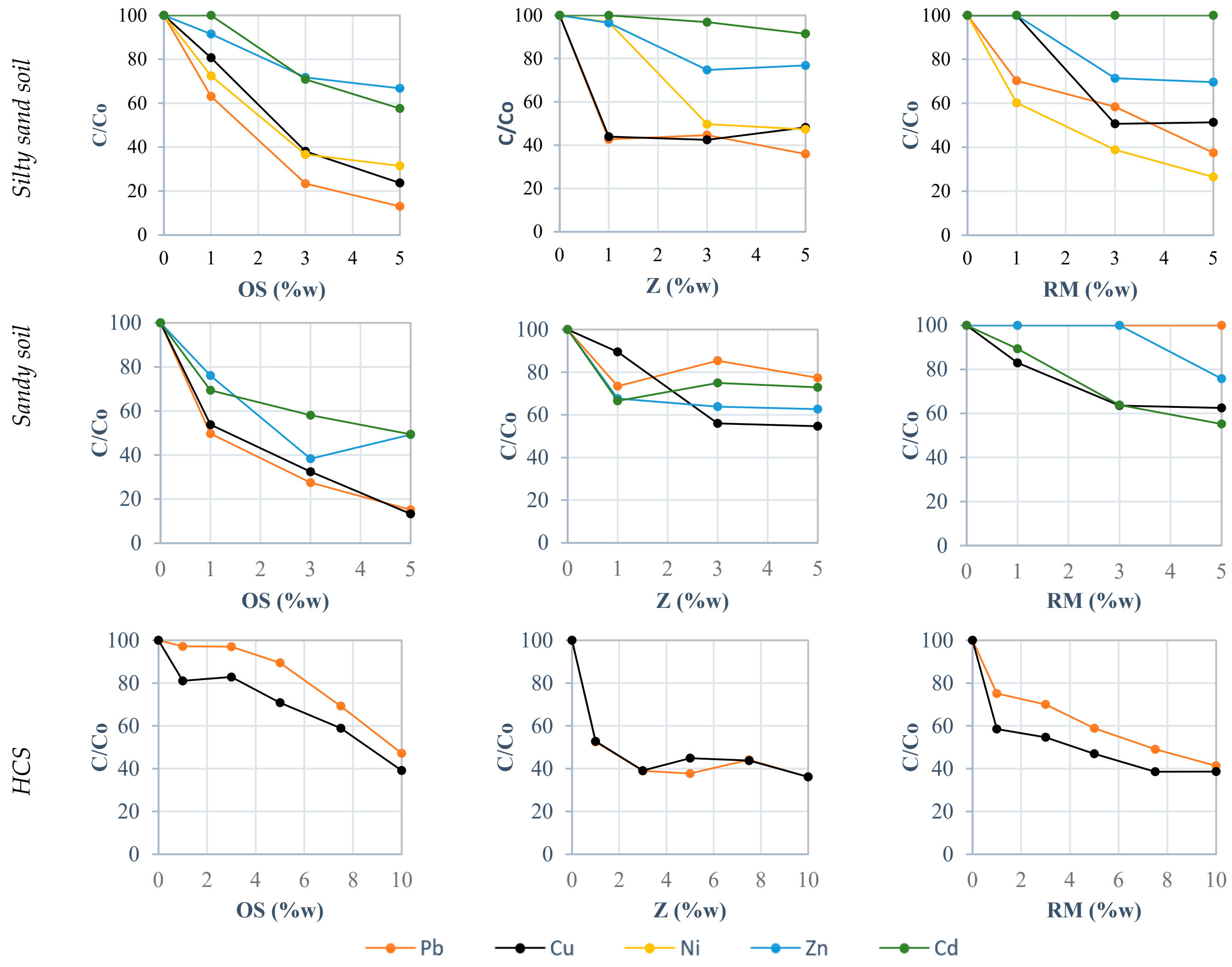

Figure S2. Normalised final toxic metal concentrations in the leachates of silty sand soil, sandy soil and HCS obtained through the TCLP test.

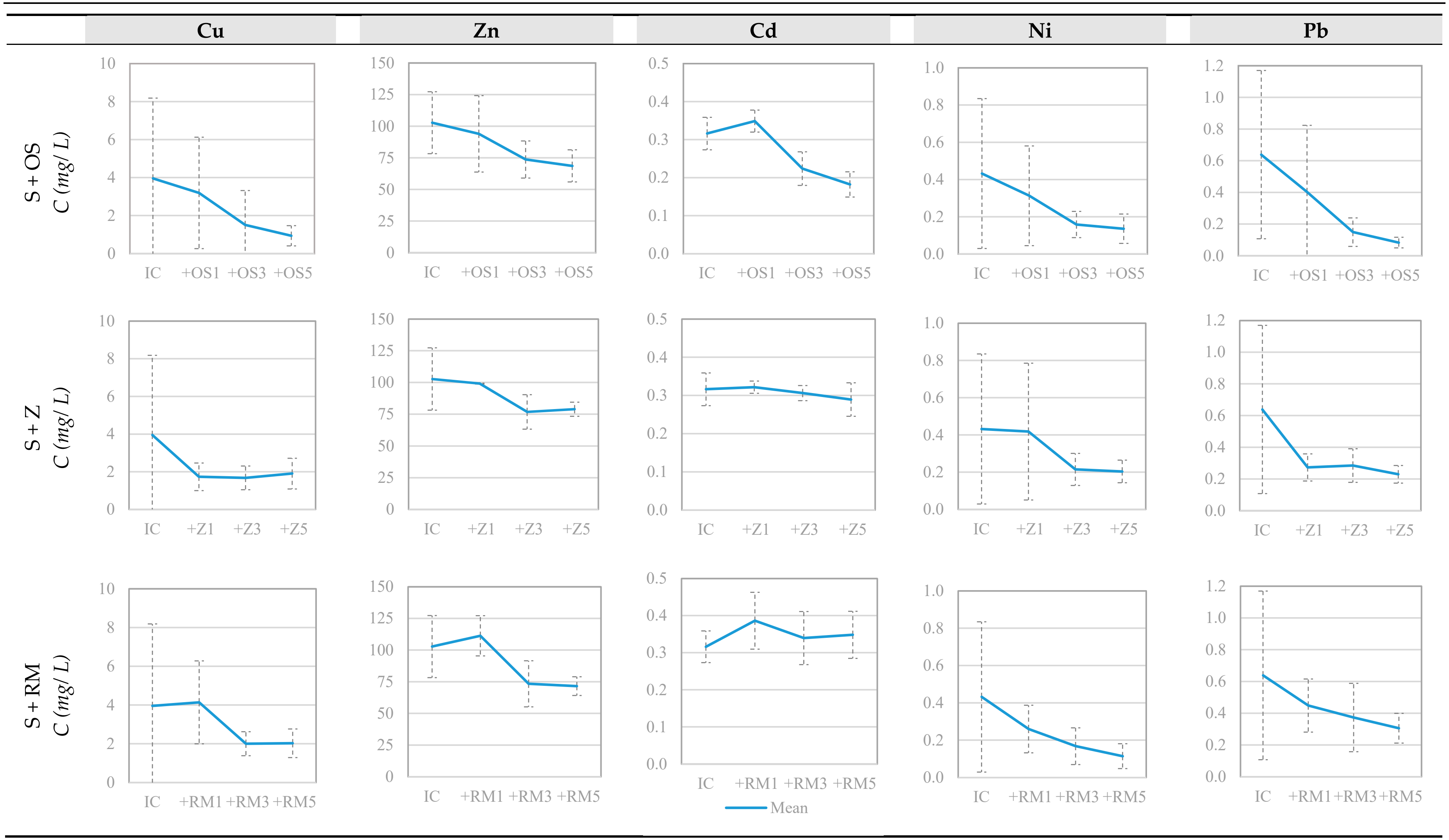

Figure S3. Variation in toxic metal concentrations in the leachate obtained via TCLP from the silty sand soil treated with OS, Z and RM. S – Soil sample; IC – Initial concentration; OS – Oyster shell; Z – zeolite; RM – Red mud. The number after the binder abbreviation represents mass percentage (e.g. Z3 means 3 wt% of Z).

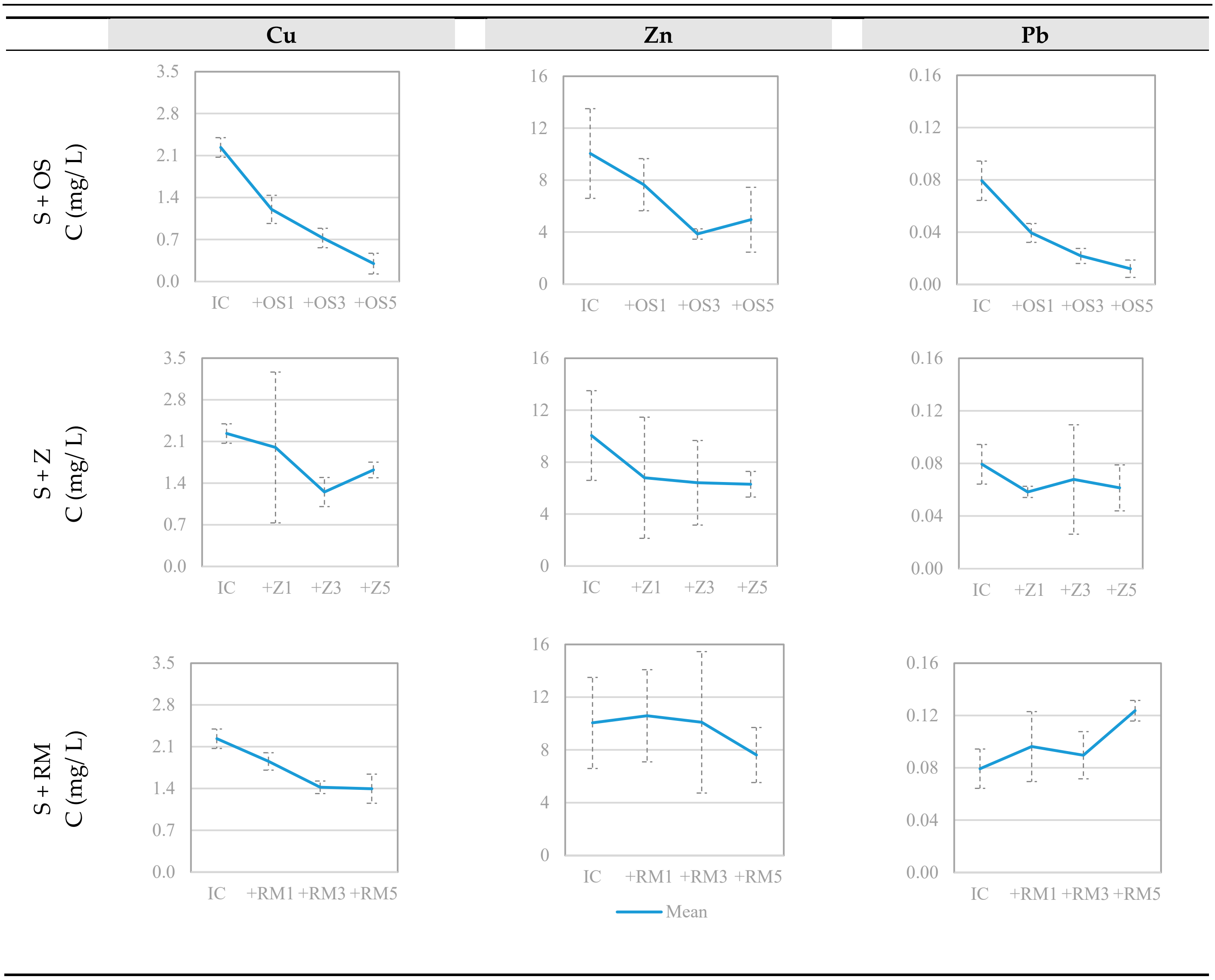

Figure S4. Variation in toxic metal concentrations in the leachate obtained via the TCLP test of the sandy soil treated with OS, Z and RM. S – Soil sample; IC – Initial concentration; OS – Oyster shell; Z – zeolite; RM – Red mud. The number after the binder abbreviation represents mass percentage.

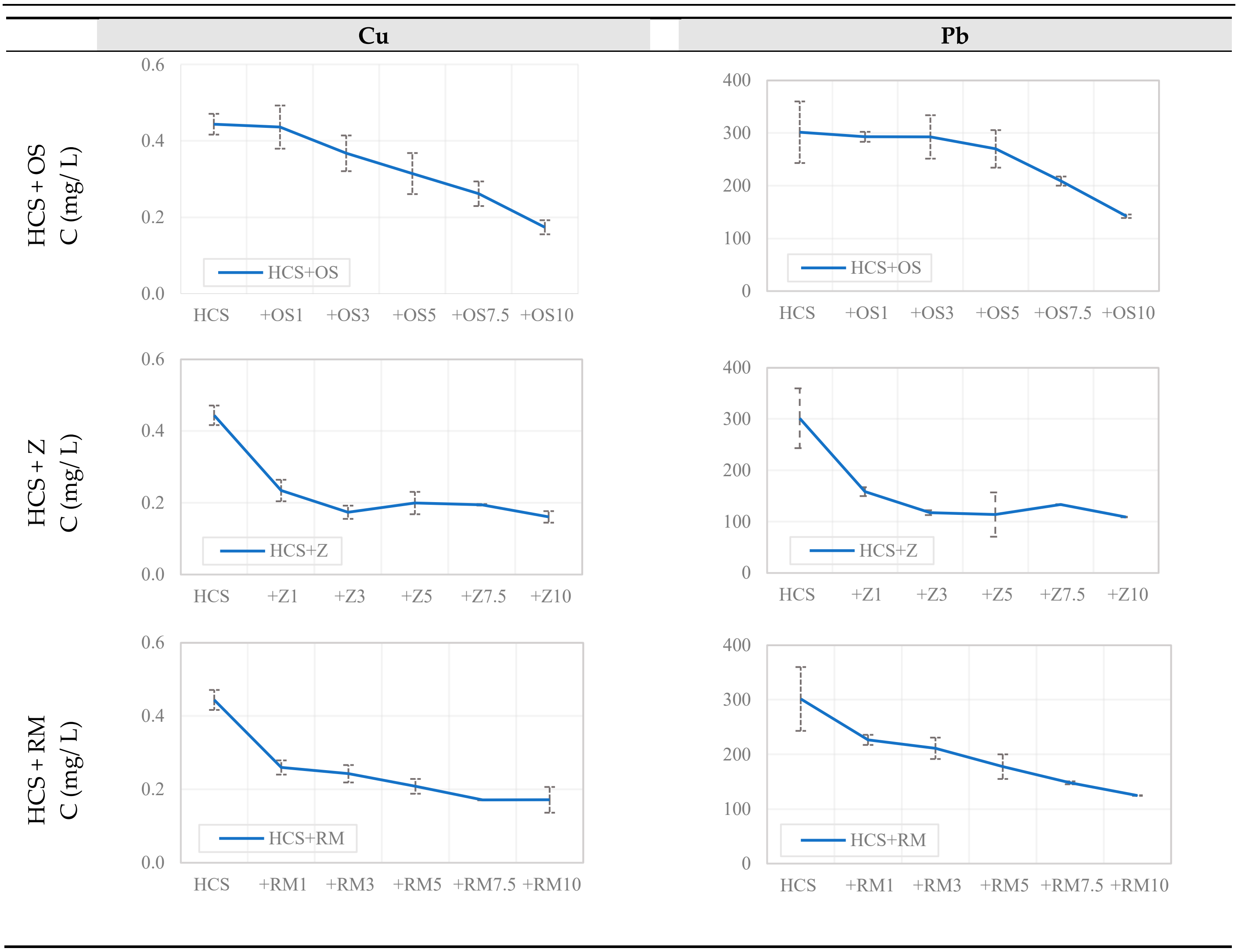

Figure S5. Variation in toxic metal concentrations in the leachate obtained via the TCLP test of the HCS treated with OS, Z and RM. OS – Oyster shell; Z – zeolite; RM – Red mud. The number after the binder abbreviation represents mass percentage (e.g. Z3 means 3 wt% of Z)

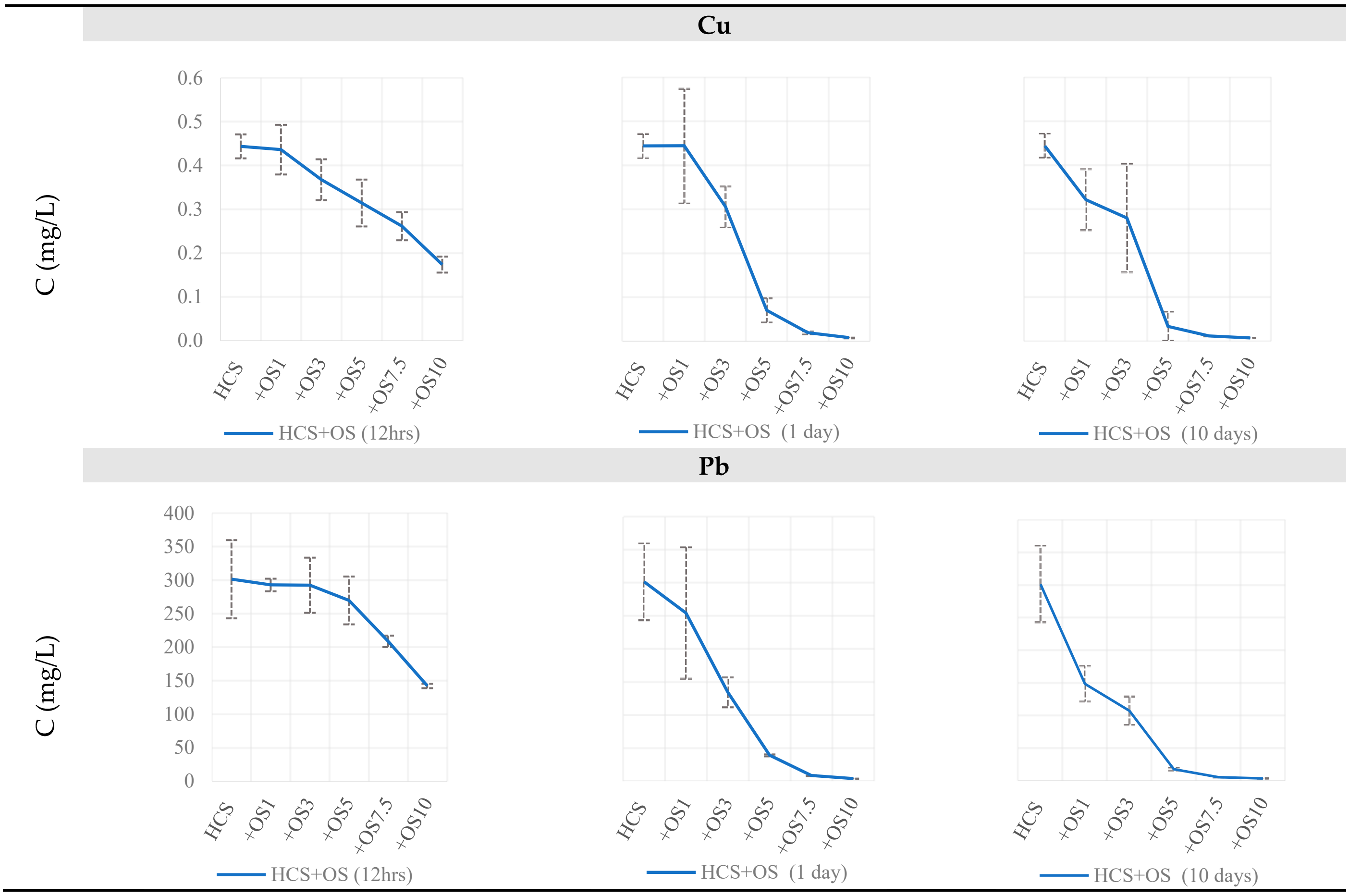

Figure S6. Pb and Cu concentrations in the leachate obtained through the TCLP test of the HCS treated with OS at different extraction (contact) times. OS – Oyster shell. The number after the binder abbreviation represents mass percentage.

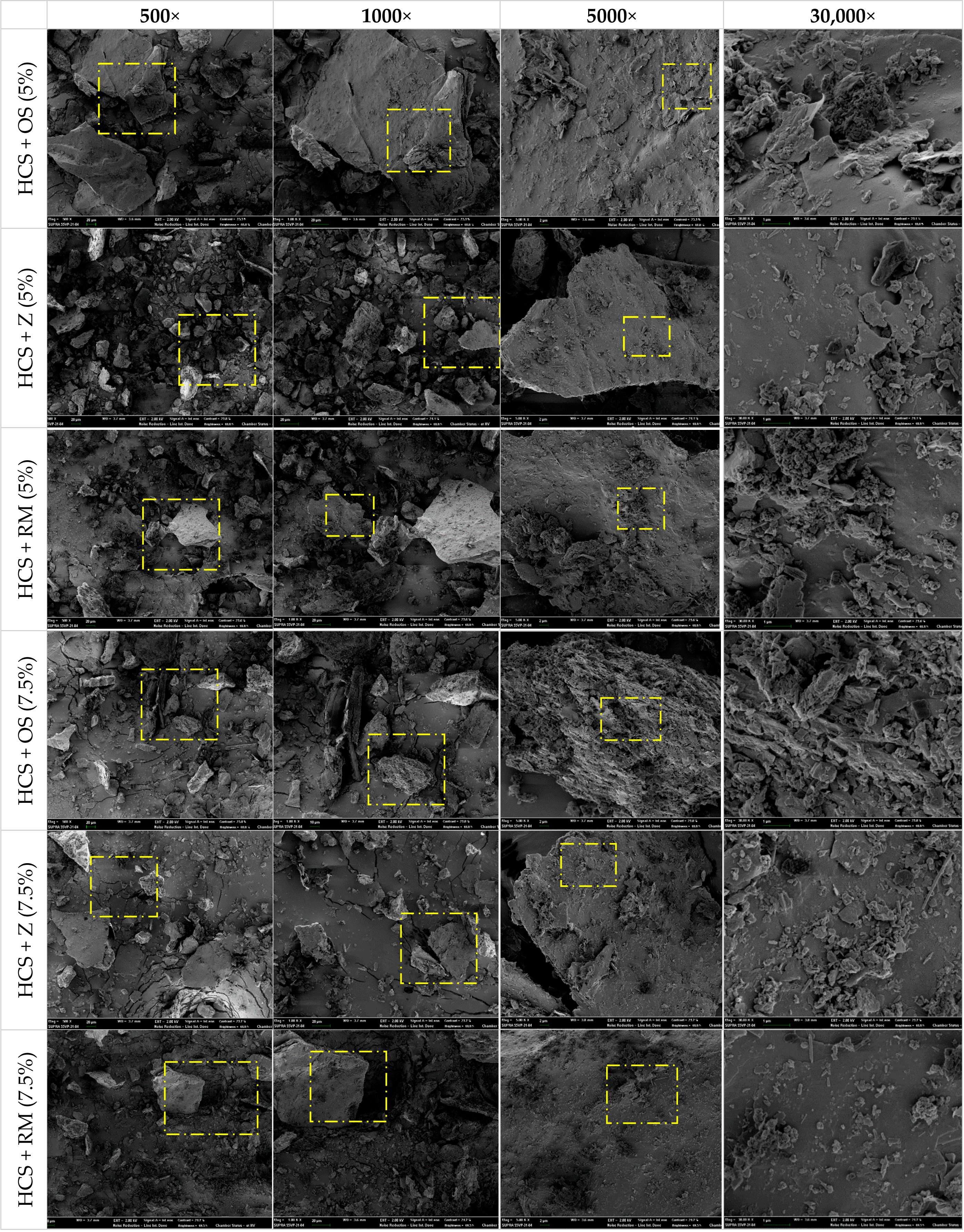
500×
1000×
5000×
30,000×
HCS + OS (5%)
HCS + Z (5%)
HCS + RM (5%)
HCS + OS (7.5%)
HCS + Z (7.5%)
HCS + RM (7.5%)

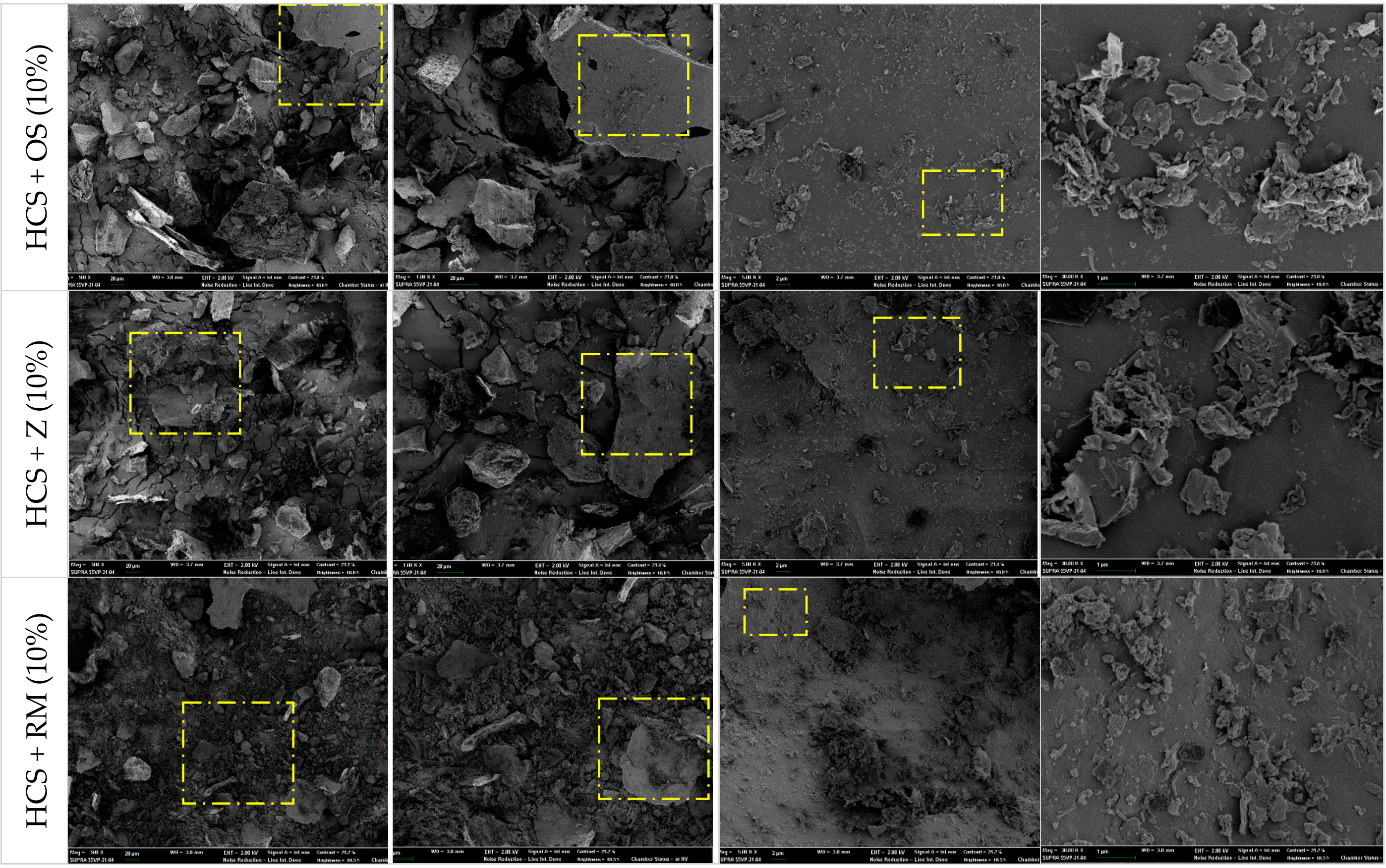

Figure S7. SEM Analysis for the sample of soil treated with each binder (5, 7.5 and 10 wt.%)
